# Designing Phenyl Porous Organic Polymers with High-Efficiency Tetracycline Adsorption Capacity and Wide pH Adaptability

**DOI:** 10.3390/polym14010203

**Published:** 2022-01-05

**Authors:** Wenjie Nie, Jiao Liu, Xue Bai, Zefeng Xing, Ying Gao

**Affiliations:** 1 College of Geology and Environment, Xi’an University of Science and Technology, Xi’an 710054, China; LJ1379885898@126.com (J.L.); baixue@shu.edu.cn (X.B.); 18409050128@stu.xust.edu.cn (Z.X.); 2 Department of Teaching Quality Evaluation, Yan’an University, Yan’an 716000, China

**Keywords:** adsorption, porous organic polymers, tetracycline, pH adaptability, reusability

## Abstract

Adsorption is an effective method to remove tetracycline (TC) from water, and developing efficient and environment-friendly adsorbents is an interesting topic. Herein, a series of novel phenyl porous organic polymers (P-POPs), synthesized by one-pot polymerization of different ratios of biphenyl and triphenylbenzene under AlCl_3_ catalysis in CH_2_Cl_2_, was studied as a highly efficient adsorbent to removal of TC in water. Notably, the obtained POPs possessed abundant phenyl-containing functional groups, large specific surface area (1098 m^2^/g) with abundant microporous structure, high pore volume (0.579 cm^3^/g), favoring the removal of TC molecules. The maximum adsorption capacity (fitted by the Sips model) could achieve 581 mg/g, and the adsorption equilibrium is completed quickly within 1 h while obtaining excellent removal efficiency (98%). The TC adsorption process obeyed pseudo-second-order kinetics and fitted the Sips adsorption model well. Moreover, the adsorption of POPs to TC exhibited a wide range of pH (2–10) adaptability and outstanding reusability, which could be reused at least 5 times without significant changes in structure and efficiency. These results lay a theoretical foundation for the application of porous organic polymer adsorbents in antibiotic wastewater treatment.

## 1. Introduction

The soaring usage of antibiotics in recent years has resulted in their widespread presence in water [1]. In particular, tetracycline (TC), as an essential antibiotic, accomplishes sterilization by inhibiting the synthesis of microbial proteins such as gram bacteria and chlamydia to control and treat diseases, and is extensively used in medication, cattle farming, and other fields due to its particular advantages, such as broad spectrum, low toxicity, low cost, and so on [2,3]. However, 30–90% of TC ingested by humans and animals is released into the aquatic environment together with excrement which is difficult to decompose [4,5]. Notably, the long-standing accumulation of TC in the environment could induce the production of bacterial resistance and even lead to the transfer of resistance genes, which further causes some serious ecological and health risk [6]. Therefore, the development of an effective method to remove TC from water circumstance is urgently necessary.

In recent decades, various methods including ion-exchange [7], membrane separation [8], chemical oxidation [9], photodegradation [10,11], adsorption [12,13,14,15] and other technologies [16,17,18] have been employed to treat TC pollution in water. Among above methods, the adsorption is widely used in the field of TC wastewater treatment due to its advantages such as simple operation, low price, random reaction conditions, no significant changes in active sites and spatial structure after adsorption, and reusability of absorbents [19,20,21]. More importantly, for adsorption, the efficiency and practical feasibility are extremely dependent on the type and nature of the adsorbent. To date, the existing adsorption materials used for the removal of TC include: clay [22], graphene oxide [23], activated carbon [24], metal organic framework compounds [25], and so on [26,27]. However, the development of excellent performance adsorbents is still challenging for TC removal, since the previously reported materials have certain disadvantages, such as lengthy procedures of preparation, weak selective adsorption capacity for TC, low adsorption rate, limited narrow pH rang, and difficulty in regeneration.

Surprisingly, due to the high porosity, large specific surface area, excellent stability, and pore structure adjustability through the introduction of specific functional groups, porous organic polymers as an advanced porous material are gaining widespread attention [28,29,30]. In recent years, the research on adsorbent-based porous organic polymers (POPs) has come to prominence because of their unique adsorption properties for antibiotics in water. For example, in 2018, Liu [31] et al. devised and synthesized diol-based porous organic polymers for the first time as absorbents for TC removal, and mechanism research showed that the multidentate hydroxyl groups of diol-based porous organic polymers ensured high binding energy to TC species. However, the maximum adsorption capacity was only 155.8 mg/g and it taken up to 300 min to reach adsorption equilibrium, and the high adsorption performance was limited to the pH 8–10 of the solution. Further development of porous organic polymers with high efficiency and conditional universality to improve the removal performance for TC is worth looking forward to. Then, Song [32] and co-workers developed conjugated microporous polymers via the Pd-catalyzed Suzuki coupling reactions of 2,4,6-tris(4-bromophenyl) pyridine with two aromatic borates and obtained excellent adsorption efficiency using it for the adsorption of TC in aqueous solution. Unfortunately, although the adsorption equilibrium in this process could be reached within 1 h and the maximum adsorption capacity of the material was as high as 909.09 mg/g, the addition of organic solvents to adjust the dispersibility of the adsorbent in water and the environmental risk of heavy metals to aquatic plants remains a challenge. Moreover, the reusability of the CMP materials still needs further exploration. Therefore, it is still pressing to design and fabricate specific, efficient, wide range of pH adaptability, environment-friendly, and recyclable POPs as adsorbents to remove TC pollution in water.

Herein, we used biphenyl and triphenylbenzene as feedstock to synthesize four kinds of phenyl porous organic polymers (P-POPs) as adsorbents to remove TC in water. In addition, the structure and property of the obtained P-POPs were characterized and analyzed by scanning electron microscopy (SEM), Brunauer–Emmett–Teller (BET), Fourier transform infrared spectroscopy (FT-IR), Thermogravimetric analysis (TGA), zeta potential analyzer, and so on. Furthermore, the affecting factors, adsorption kinetics, and adsorption isotherms of TC adsorption were systematically investigated. The stability and reusability of P-POPs were also evaluated. The optimized P-POPs was possessed high TC adsorption amount of 581 mg/g, fast adsorption kinetics (about 1 h of adsorption equilibrium time), wide range of pH adaptability (no significant difference in TC adsorption capacity in pH 2–10 range), excellent recyclability (be reused at least 5 times without significant changes in their structure), good dispersion performance in aqueous solutions and no need for organic solvents. This work is significant to the exploration of new adsorbents, and provides certain theoretical support for the adsorption of porous organic polymers (POPs) to eliminate antibiotic pollution.

## 2. Materials and Methods

### 2.1. Chemical Reagents

The materials used in this work are all analytically pure (AR), and unless otherwise specified, they will not be processed and used directly. Biphenyl (99%), triphenylbenzene (98%) and anhydrous aluminum trichloride (99%) were purchased from Saen Chemical Technology Co., Ltd. (Shanghai, China). Dichloromethane, methanol, and tetrahydrofuran were supplied by Shanxi Tongjie Chemical Reagent Co., Ltd. (Shanxi, China). Sodium hydroxide was purchased from Guangdong Guanghua Sci-Tech Co., Ltd. (Guangdong, China).

### 2.2. Typical Procedure for the Synthesis of P-POPs

**Scheme 1:**
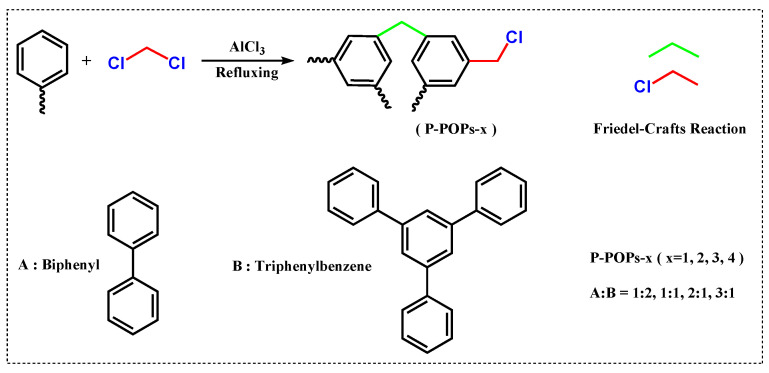
The synthesis route of phenyl porous organic polymers (P-POPs).

Four P-POPs were prepared on the basis of the previous reports with certain adjustments, and the typical synthetic pathway was illustrated in Scheme 1 [30]. Four kinds of P-POPs were synthesized from biphenyl and triphenylbenzene in different molar ratios (biphenyl:triphenylbenzene = 1:2, 1:1, 2:1, 3:1, respectively) as monomers, and they were sequentially expressed as P-POPs-x (x = 1~4). Taking the preparation of P-POPs-1 samples as an instance, the reaction was run in a 100 mL double-necked flask equipped with a condenser under the protection of nitrogen. First, anhydrous aluminum trichloride (1.06 g) was added into the reactor containing 60 mL of dichloromethane and stirred for 1 h; then 77 mg of biphenyl (0.5 mmol) and 306 mg of triphenylbenzene (1 mmol) were quickly added to the reaction flask, and immediate discoloration was observed. After refluxing at 70 °C for 16 h, the mixture was filtered and washed with methanol/dilute hydrochloric acid (1:1 volume ratio) three times to removed possible residual aluminum trichloride. Afterwards, the product was further refluxed and washed for 48 h through Soxhlet extractor with tetrahydrofuran/Methanol/water (1:1:1 volume ratio). Finally, the P-POPs-1 polymer material was obtained by vacuum drying at 130 °C for 12 h [30].

### 2.3. Characterization of Samples

The surface morphologies of P-POPs were recorded by scanning electron microscopy (SEM, JSM-7610F, Electronics Corporation, Tokyo, Japan). The specific surface area and pore-size distribution of the P-POPs were evaluated based on the Brunauer–Emmett–Teller method (BET, Micromeritics, Atlanta, GA, USA). The determination of the chemical structure was done by Fourier transform infrared spectroscopy (FT-IR, TENSOR 27, Bruker, Karlsruhe, Germany). The thermal stability of the materials was detected by thermogravimetric analysis (TGA, STA PT1600, Linseis, Bavaria, Germany). The surface potential values of P-POPs-3 were measured by zeta potential analyzer (Malvern Instrument Co., Malvern, UK).

### 2.4. Adsorption Experiment

All experiments were carried out to explore the adsorption performance of P-POPs at 25 °C. The 40 mL of 50 mg/L TC solution was added into the centrifuge tube containing 0.01× *g* P-POPs. Then the centrifuge tube was placed in a gas bath to shake for 7 h at 150 rpm under dark to ensure adsorption equilibrium. After adsorption, the concentrations of residual TC in the filtrate were detected by an UV-vis spectrophotometer (UV 2600, Shmadezu, Japan, λ = 356 nm). All the adsorption experiments were conducted in triplicates. In addition, target uptake at equilibrium (q_e_, mg/g) and corresponding removal rate (R_r_, %) were obtained from the follow Equations (1) and (2), successively:

(1) $$q_{e}=\frac{\left(C_{0\;}{-\;C}_{e}\right)V}{m}$$

(2) $$R_{r}=\frac{C_{0}{\;-\;C}_{e}}{C_{0}}\;\times \;100\%\;$$

where C_0_ (mg/L) and C_e_ are the initial and equilibrium concentration of TC. V (L) means the volume of TC solution and m (g) represents the P-POPs dosage.

To study the adsorption kinetics, the adsorption process was carried out using 50 mg/L TC solution at a time interval of 5–420 min. The equations of pseudo-first- and pseudo-second-order kinetics are expressed as follows Equations (3) and (4):

(3) $$\ln\left(q_{e\;}{-\;q}_{t}\right){=\mathrm{lnq}}_{e}{\;-\;k}_{1}t\;$$

(4) $$\frac{t}{q_{t}}=\frac{1}{k_{2}q_{e}^{2}}+\frac{t}{q_{e}}\;$$

where q_t_ and q_e_ (mg/g) are the quantity of TC adsorbed onto P-POPs at time t and at equilibrium (min), successively, and k_1_ (1/min) and k_2_ (g/(mg∙min)) are the rate constants of the pseudo-first-order adsorption process and pseudo-second-order adsorption process, successively.

To investigate the effect of adsorbent dosage and solution pH, the dosage of P-POPs-3, the optimal material previously screened out, was varied in the range of 4–20 mg, and the pH value of TC solution increased from 2 to 12 under the adjustment of 0.01 mol/L HCl and NaOH.

The adsorption isotherm was investigated through varying the initial concentration of TC solution from 10 to 270 mg/L. The Langmuir, Freundlich, and Sips equations were used to fit the experimental data. In addition, the Langmuir isotherms Equation (5), the Freundlich isotherms Equation (6) and Sips isotherms Equation (7) are as follows:

(5) $$q_{e}=\frac{q_{m}k_{L}C_{e}}{{1+k}_{L}C_{e}}$$

(6) $$q_{e}{=k}_{F}C_{e}^{n}$$

(7) $$q_{e}=\frac{q_{e}{\left({\mathrm{bC}}_{e}\right)}^{1/n}}{1+{\left({\mathrm{bC}}_{e}\right)}^{1/n}}$$

where q_e_ (mg/g) is the amounts of TC adsorbed at equilibrium, C_e_ (mg/L) is the equilibrium concentration of TC onto the adsorbent; q_m_ (mg/g) is the maximum monolayer adsorption, k_L_ is Langmuir adsorption constant; k_F_ is Freundlich constant representing for adsorption capacity and n is the heterogeneity factor; 1/n is also the heterogeneity factor and its close to 1 means the relatively uniform surface of the adsorbent, b (L/mg) represents the median association constant.

In addition, the adsorbed P-POPs-3 was desorbed and regenerated using 50 mL 0.2 M NaOH as the eluent to desorb and regenerate 10 mg of TC-adsorbed P-POPs-3 under ultrasonic conditions for 30 min, and finally dry at 130 °C for 12 h [33]. Then, the regenerated P-POPs-3 was used as adsorbent and recycled at 25 °C. The cyclic adsorption-desorption test was run continuously for 5 times. In addition, the obtained desorption rates of the first to fifth cycles were 92%, 90%, 89%, 90%, and 87%, respectively.

## 3. Results and Discussion

### 3.1. Structural Analysis and Characterization of Material

**Figure 1:**
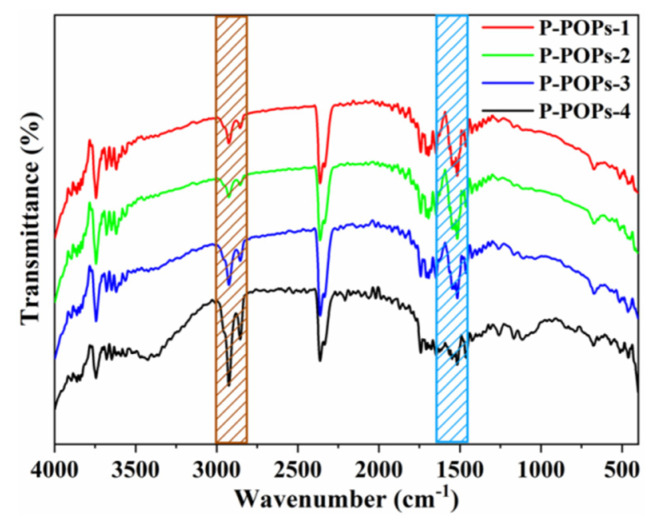
FT-IR spectrum of P-POPs-1, P-POPs-2, P-POPs-3, P-POPs-4.

The FT-IR analysis of P-POPs is shown in Figure 1. The adsorption peak appeared at 1460–1650 cm^−1^ reveals the existence of the benzene ring structure. The obvious bond at 2810–3010 cm^−^^1^ was corresponding to the absorption peak of C–H, which could be attributed to the stretching vibration of the methylene group (–CH_2_–). This results indicated that the four kinds of P-POPs synthesized by Friedel-Crafts reaction between the raw materials and dichloromethane, which were consistent with the previous reports [30,34].

**Figure 2:**
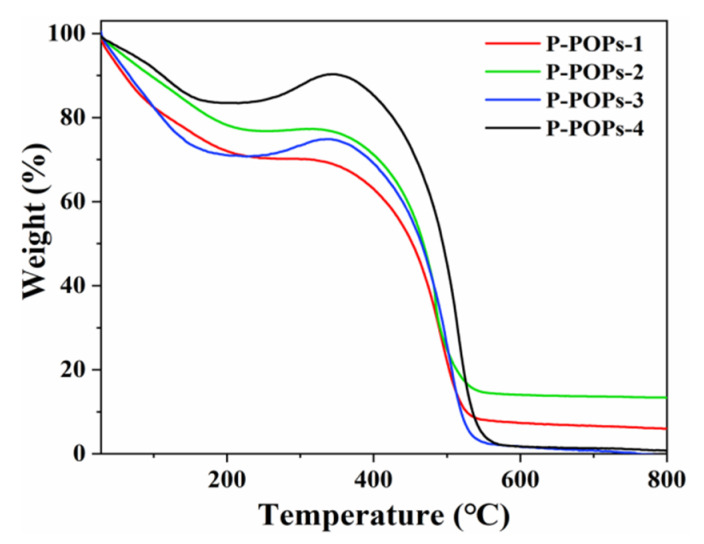
Thermogravimetric analysis of P-POPs under air.

The synthesized P-POPs samples were insoluble in a series of common organic solvents such as dichloromethane, methanol, ethanol, ethyl acetate and tetrahydrofuran, indicating their high chemical stability. Moreover, to prove the thermostability of P-POPs, thermogravimetric analysis (TGA) curves are shown in Figure 2. A slight weight loss of about 15~30% from 0 to 200 °C for P-POPs, which may be corresponded to the loss of residual solvent in the polymers or the escape of adsorbed water vapor [35]. At around 330 °C, the weight increases on the thermogravimetric curve possibly due to a small amount of aluminum resulting from the AlCl_3_ catalyst remained in the polymers, which may be converted into Al_2_O_3_ or other Al oxides after burning in the air. Importantly, P-POPs had no significant degradation below 330 °C and the dramatic weight loss was about 450 °C, which indicated their high thermal stability.

**Figure 3:**
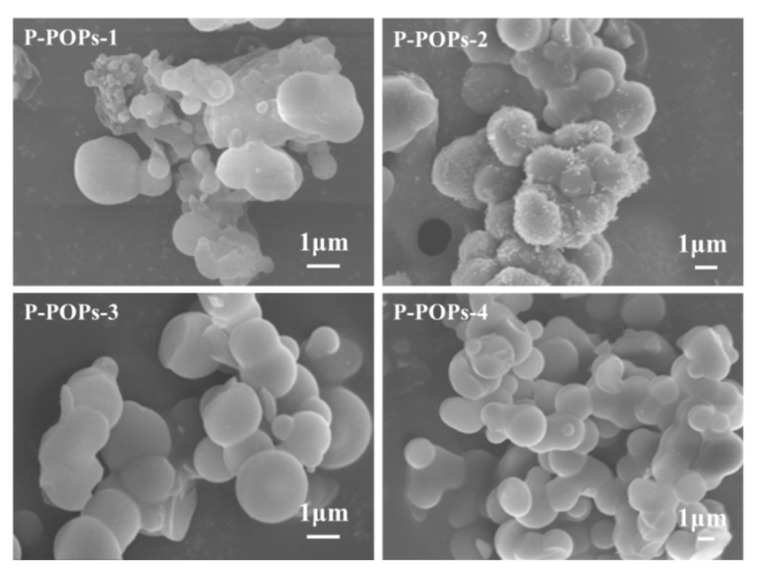
The SEM images of P-POPs.

The different morphologies of P-POPs were observed by a scanning electron microscope (Figure 3). P-POPs-1 and P-POPs-4 were comprised of a detached-spherical morphology, while P-POPs-2 and P-POPs-3 were comprised of a fused-spherical morphology. In addition, the average diameters of P-POPs computed by NanoMesurer software were 0.39, 4.68, 0.48, and 1.54 μm, respectively.

**Figure 4:**
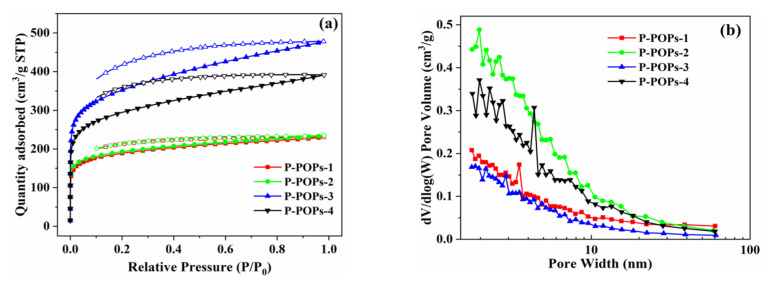
(**a**) N_2_ adsorption-desorption isotherms measured at 77 K and (**b**) pore-size distribution curves for P-POPs.

Figure 4a showed the N_2_ adsorption and desorption isotherms of P-POPs at 77 K. It was clear that all the four P-POPs exhibited type I sorption isotherms, which was one of the main characteristics of microporous materials. The non-occlusive nature of the adsorption/desorption isotherm may be due to the expansion of the polymer matrix, which was common in porous polymer. In addition, the porosity properties of P-POPs are listed in Table 1, their BET specific areas calculated from adsorption data were 571, 580, 1098, 908 m^2^/g for P-POPs-1, P-POPs-2, P-POPs-3, P-POPs-4, respectively. Clearly, the synthesized P-POPs had excellent specific surface area, and the highest P-POPs-3 reached 1098 m^2^/g, indicating that the use of biphenyl and triphenylbenzene as raw materials was beneficial to the synthesis of organic polymer materials with higher specific surface area, and the ratio of biphenyl with triphenylbenzene is an important factor affecting the specific surface area of the polymer. Moreover, P-POPs-3 possess the largest pore volume and median pore size, which are indispensable factors leading to its excellent adsorption performance. On the other hand, the pore-size distribution of P-POPs were displayed in Figure 4b. The pore width of P-POPs were mainly concentrated in the microporous area, which contributed to the excellent adsorption performance for TC, since the total molecule length of TC is 1.27 nm [21].

### 3.2. Effect of Adsorbent

**Figure 5:**
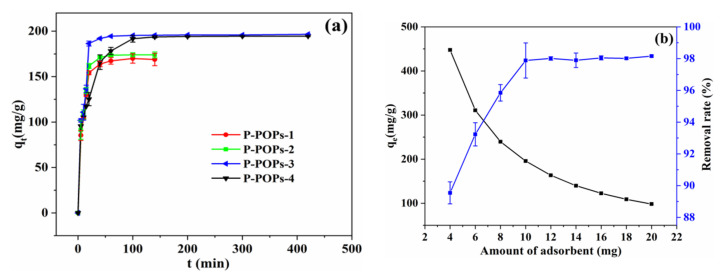
(**a**) The influence of adsorbent type and (**b**) dosage.

The TC removal performance of different P-POPs were evaluated by adding 10 mg of absorbents to 40 mL of 50 mg/L TC aqueous solution (Figure 5a). In the first 20 s, the adsorption amount of TC by the four P-POPs increased rapidly with time, and then gradually flattened until the adsorption equilibrium. Notably, with the same initial concentration, the adsorption capacities of P-POPs for TC were in the sequence of P-POPs-3 (196 mg/g) > P-POPs-4 (195 mg/g) > P-POPs-2 (174 mg/g) > P-POPs-1 (170 mg/g). It can be concluded that the highest TC adsorption capacity (196 mg/g) was observed via P-POPs-3, which was consistent with the results on the specific surface area. This phenomenon indicated that the specific surface area is important factor affecting the adsorption performance of P-POPs. Then, P-POPs-3 was selected as the optimal materials to optimize the number of absorbents, and the influence of the amount of absorbent on the adsorption effect in the range of 4–20 mg was studied (Figure 5b). When the dosage of P-POPs was in the range of 4 mg to 10 mg, the removal rate of TC was positively correlated with the amount of adsorbent. This was because the increase in the amount of adsorbent in the solution enhanced the collision probability of P-POPs particles with TC molecules, therefore improving its adsorption efficiency of TC in the aqueous solution. However, when the amount of adsorbent was further increased, the removal efficiency did not change significantly. Therefore, 10 mg of adsorbent was selected for subsequent experiments, and the corresponding removal rate was 98%. On the other hand, the adsorption capacity was negatively correlated with the amount of adsorbent, which may be caused by the discordant ratio between the number of TC molecules and the vacant sites of the P-POPs.

### 3.3. Adsorption Kinetics

**Figure 6:**
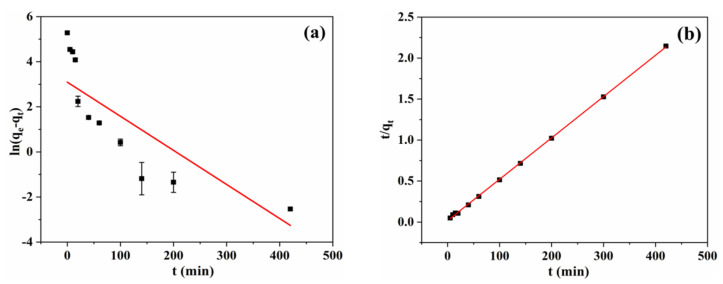
The fitted kinetics curves for TC adsorption onto P-POPs-3 by (**a**) pseudo-first-order model and (**b**) pseudo-second-order model.

Here, the adsorption kinetics of TC on P-POPs were illustrated in Figure 6. It was found that the TC adsorption was soared in the first 40 min, which may be due to abundant adsorption sites and pores. Then, with the saturation of TC on the surface of the adsorbent and its active sites, the adsorption capacity increased slowly and reached equilibrium in about 60 min. Two widely used kinetic models, namely pseudo-first-order and pseudo-second-order kinetics, were applied to evaluate the adsorption kinetics. Notably, the fitting curve generated by the pseudo-second-order kinetic model could describe the experimental data more closely and present a higher R^2^ value (0.999) (Table 2). On the other hand, the q_max_ of the pseudo-second-order kinetic model was closer to the experimental data (q_exp_) (Table 2). Thus, the adsorption of TC by P-POPs obeyed pseudo-second-order kinetics, which indicated that chemical adsorption was the main adsorption mechanism.

### 3.4. Adsorption Isotherms

**Figure 7:**
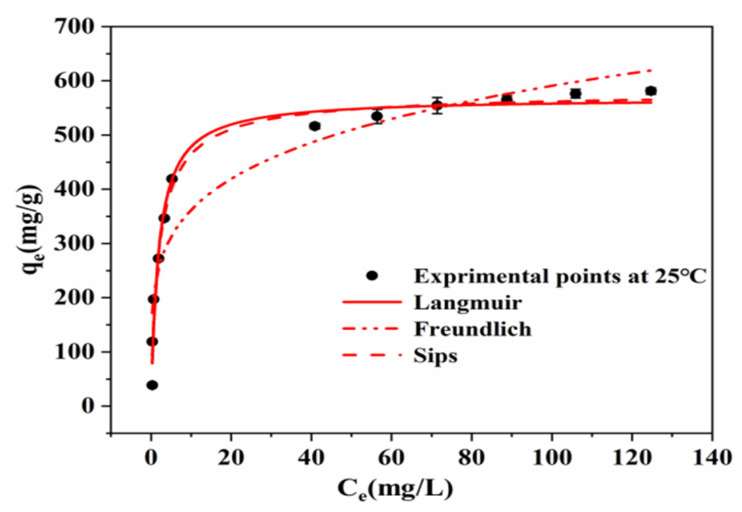
The experimental and the fitted isothermal adsorption curves for TC on P-POPs-3 by Langmuir, Freundlich and Sips models at 25 °C, respectively.

As shown in Figure 7, the effect of TC mass concentration on the adsorption capacity was investigated by adding 10 mg P-POPs to 10~270 mg/L TC solution. When the initial concentration of TC increased from 10 mg/L to 170 mg/L, the adsorption capacity of TC on P-POPs enhanced rapidly, and then, the growth rate decreased and tended to balance. The adsorption behavior of TC onto P-POPs was assessed by the Langmuir, Freundlich and Sips isotherm models, and the correlation coefficients (R^2^) were 0.9829, 0.9078 and 0.9850, respectively (Table 3). The results showed that the Sips model had a higher R^2^ value and the theoretical maximum adsorption capacities (581 mg/g) calculated from Sips model were approximated to the experimental date (581 mg/g) (Table 3). In addition, the value of 1/n obtained by the Sips fitting was close to 1, indicating that the surface of the adsorbent was relatively uniform, and the Sips isotherm was close to the Langmuir equation, which was consistent with the fitting results in the figure. On the other hand, the 1/n value obtained in the Freundlich model is less than 1.0, indicating that the adsorbent was beneficial to remove TC from the aqueous solution.

### 3.5. The Effect of pH

**Figure 8:**
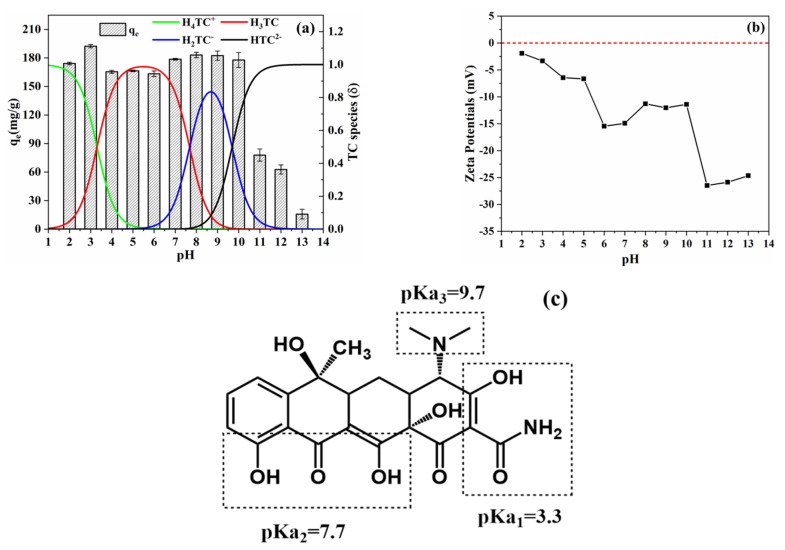
(**a**) The pH effect on TC adsorption, (**b**) zeta potential of P-POPs-3 and (**c**) the molecule structure and ionization constants of TC.

The solution pH was an important factor affecting the interaction between the adsorbent and the adsorbate. As shown in Figure 8a, P-POPs-3 showed an obvious pH-dependent adsorption mode for TC. Figure 8b showed the zeta potential at different pH values, which was clear that the P-POPs-3 was positively charged in the pH range of 2–14. When the pH of the solution was in the range of 2–8, the adsorption capacity of TC was maintained at a high level and showed the optimal result at pH 3, indicating that lower pH had a positive effect on the adsorption of TC in P-POPs-3. This phenomenon may be due to the presence of TC in the form of cations at pH < 3.3 [35]. Compared with pH 3, the adsorption capacity at pH 2 was slightly lower, which probably because the high concentration of H^+^ leads to stronger competition at the adsorption site than electrostatic attraction. When the pH of the solution was in the range of 4–10, the adsorption capacity showed a small difference and decreased compared with pH 3. This result may be because TC was zwitterion (H_3_TC) and anions (H_2_TC^−^ and HTC^2−^) at varying pHs from 3.3 to 7.7 and pH > 7.7 (Figure 8c) [36], respectively, and the addition of anions offset part of the electrostatic attraction. The significant decrease in adsorption performance at pH > 10 may be attributed to the fact that P-POPs-3 and TC molecules had the same charge, causing in electrostatic repulsion and a significant reduction in the TC adsorption capacity. As mentioned above, the variations of adsorption capacity at different pH indicated that the effect of electrostatic attraction was contributed to TC adsorption. In addition, there was abundant π-π interaction between the benzene ring of P-POPs and the phenyl ring of TC, which drives chemical adsorption. Therefore, electrostatic attraction and π-π interaction mainly favor the efficient adsorption of TC.

### 3.6. Reused of the Adsorbents

**Figure 9:**
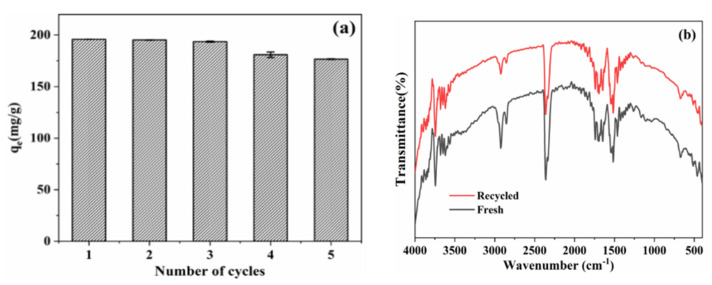
(**a**) The reusability of the P-POPs for the removal of TC and (**b**) FT-IR spectra of the recycled P-POPs-3.

To evaluate the stability and reusability of P-POPs-3, (P-POPs-3)-TC was desorbed by 0.2 M NaOH solution, which was based on the weak correlation with TC adsorption under alkaline conditions. After three times of adsorption-desorption cycles, the performance of the regenerated adsorbent did not show a significant change (the adsorption capacities are 196, 195, and 194 mg/g, respectively.) (Figure 9a). In the further cycles, the adsorption efficiency exhibited a slight drop, but fortunately, the adsorption capacity could still reach 176 mg/g (88%) after five cycles, which proved the excellent regeneration of P-POPs-3 in removing TC from the aqueous solution. To further support the result, the recycled P-POPs-3 was detected by FT-IR and shown in Figure 9b. Obviously, the characteristic peaks of the recycled P-POPs-3 were almost the same as the fresh adsorbent, and no obvious structural change was occurred during the process of adsorption and desorption, further indicating that the P-POPs were highly stable and excellently recyclable.

## 4. Conclusions

In conclusion, a series of novel phenyl porous organic polymers with excellent adsorption properties were prepared through the AlCl_3_-catalyzed one-pot polymerization. The morphology, structure, and stability of as-prepared P-POPs were studied by different characterization testing technologies. In particular, abundant phenyl-containing functional groups, large specific surface area (1098 m^2^/g) with abundant microporous structure, high pore volume (0.579 cm^3^/g) made it exhibit favorable adsorption performance to the bulky TC molecule. The adsorption equilibrium was achieved within 60 min, the removal rate of TC (50 mg/L) could reach 98%, and the corresponding adsorption capacity was 196 mg/g. Moreover, the highly efficient TC adsorption could be achieved in a wide pH range (2–10) and reused at least 5 times without significant changes in structure and efficiency. We proposed that electrostatic attraction and π-π interaction were responsible for the adsorption of TC onto P-POPs. Due to the outstanding structure properties, favorable adsorption ability, wide pH adaptability, and good reusability, P-POPs will promise great potential in removing TC pollution from aqueous solution.

## Figures and Tables

**Table 1:** Information on the surface areas, pore volumes, and median pore diameter of P-POPs.

Sample	S_BET_ (m^2^/g)	V_max_ (cm^3^/g)	W_med_/nm
P-POPs-1	571	0.305	0.678
P-POPs-2	580	0.311	0.637
P-POPs-3	1098	0.579	0.698
P-POPs-4	908	0.481	0.685

**Table 2:** Kinetic parameters for the pseudo-first-order kinetic model and pseudo-second-order kinetic model.

q_exp_ (mg/g)	Pseudo-First-Order			Pseudo-Second-Order		
	k_1_ (1/min)	q_max_ (mg/g)	R^2^	k_2_ (g/(mg·min))	q_max_ (mg/g)	R^2^
196	0.01512	9.0	0.639	0.0014	198.4	0.999

**Table 3:** Parameters obtained from the Langmuir, Freundlich, and Sips models.

Langmuir Model			Freundlich Model			Sips Model			
q_m_ (mg/g)	k_L_ (L/mg)	R^2^	k_F_ (mg/g)	n	R^2^	q_m_ (mg/g)	b (L/mg)	n	R^2^
568	0.5297	0.9829	221.3034	4.6906	0.9078	581	0.5833	1.1480	0.9850
